# MicroRNA-146a suppresses rheumatoid arthritis fibroblast-like synoviocytes proliferation and inflammatory responses by inhibiting the TLR4/NF-kB signaling

**DOI:** 10.18632/oncotarget.24050

**Published:** 2018-01-08

**Authors:** Wei Liu, Yuan-Hao Wu, Lei Zhang, Bin Xue, Yi Wang, Bin Liu, Xiao-Ya Liu, Fang Zuo, Xiao-Yan Yang, Fu-Yu Chen, Ran Duan, Yue Cai, Bo Zhang, Yang Ji

**Affiliations:** ^1^ Department of Rheumatology and Immunology, First Teaching Hospital of Tianjin University of Traditional Chinese Medicine, Tianjin 300193, China; ^2^ Institute of Basic Research in Clinical Medicine, China Academy of Chinese Medical Sciences, Beijing 100700, China; ^3^ The 272nd Hospital of Chinese People's Liberation Army, Tianjin 300020, China

**Keywords:** microRNA-146a, proinflammatory cytokine, fibroblast-like synoviocytes, rheumatoid arthritis, TLR4/NF-κB signaling pathway

## Abstract

This study investigated whether microRNA-146a (miR-146a) mediating TLR4/NF-κB pathway affected proliferation and inflammatory responses of rheumatoid arthritis fibroblast-like synoviocytes from 12 RA patients (RA-FLSs). FLSs in the logarithmic growth phase were assigned into the control, miR-146a mimic miR-146a inhibitor, Tak-242 (treated with TLR4/NF-κB pathway inhibitor) and mimic + lipopolysaccharide (LPS) groups. Cell proliferation and apoptosis were detected using CCK-8 assay and flow cytometry. The expression of miR-146a, TLR4/NF-κB pathway-related proteins and cytokines were determined by RT-qPCR, western blotting and ELISA, and the release of NO by Greiss reaction. RA rat models were constructed and the primary cells were classified into the control, negative control (NC), miR-146a mimic, miR-146a inhibitor, Tak-242, mimic + LPS, and TLR4 groups. Immunohistochemistry was used to detect the expression of proliferating cell nuclear antigen (PCNA) and intercellular adhesion molecular-1 (ICAM-1). The results showed that miR-146a levels were lower in RA-FLSs than control fibroblasts. miR-146a mimic and Tak-242 decreased RA-FLS proliferation and increased RA-FLS apoptosis, while miR-146a inhibitor had an opposite trend. miR-146a mimic and Tak-242 also decreased expression of TLR4, NF-κB, IL-1β, IL-6, IL-8, IL-17, COX-2, MMP-3, Seprase, and iNOS, as well as reduced NO level in RA-FLSs while miR-146a inhibitor and TLR4 increased them. TLR4 and NF-κB levels and the positive rates of PCNA and ICAM-1 expressions were lower in RA-FLSs from RA rats given miR-146a mimic from control or miR-146a inhibitor-treated rats. These results suggest that miR-146a inhibits the proliferation and inflammatory response of RA-FLSs by down-regulating TLR4/NF-κB pathway.

## INTRODUCTION

Rheumatoid arthritis (RA) is a systemic autoimmune disease characterized by chronic inflammation of the synovial joints [1]. Nearly 0.5–1% of the world population is affected by RA and the prevalence has increased over time [2]. Early intervention with anti-inflammatory drug therapy improves quality of life and clinical outcomes of RA patients [3]. However, the pathogenesis of RA remains unclear. It is commonly characterized by chronic inflammatory proliferation of the synovial linings of diarthrodial joints and associated with aberrant humoral and cellular immunity [4].

MicroRNAs (miRs) are non-coding RNAs that are approximately 22 nucleotides long, which are involved in various physiological processes like apoptosis, cell differentiation, and immune defense [5]. miR-146a inhibits cell proliferation in several cancers [6]. Moreover, the miRNAs also play a role in RA pathology. miR-346 suppresses RA progression by inhibiting the release of pro-inflammatory factors [7]. miR-146a represses cartilage and synovial inflammation [8, 9]. MicroRNAs modulate granulocyte activation, lymphoid B cell maturation, and toll-like receptor (TLR) signaling pathways that lead to generation of pro-inflammatory cytokines [10–12]. miR-146a is induced in response to TLR4 signaling in monocytes that play a key role in innate immune response [13]. Taganov *et al.* reported that the induction of miR-146a is regulated by nuclear factor-kappa B (NF-κB) [14]. Recent studies explored the role of TLR4/NF-κB signaling pathway in RA [15]. SHH-Gli signaling pathway inhibitor clarithromycin inhibits FLS proliferation in RA patients [16]. Moreover, it has been demonstrated that miR-146a expression was higher in RA-FLSs when compared with those from patients with osteoarthritis (OA)-FLSs [17]. Based on the research of the combined effects of miR-146a and TLR4 in RA, we investigate the role of miR-146a and TLR4/NF-κB signaling pathway in inflammatory response and proliferation of FLSs in RA.

## RESULTS

### mir-146 expression in RA patients

**Figure 1:**
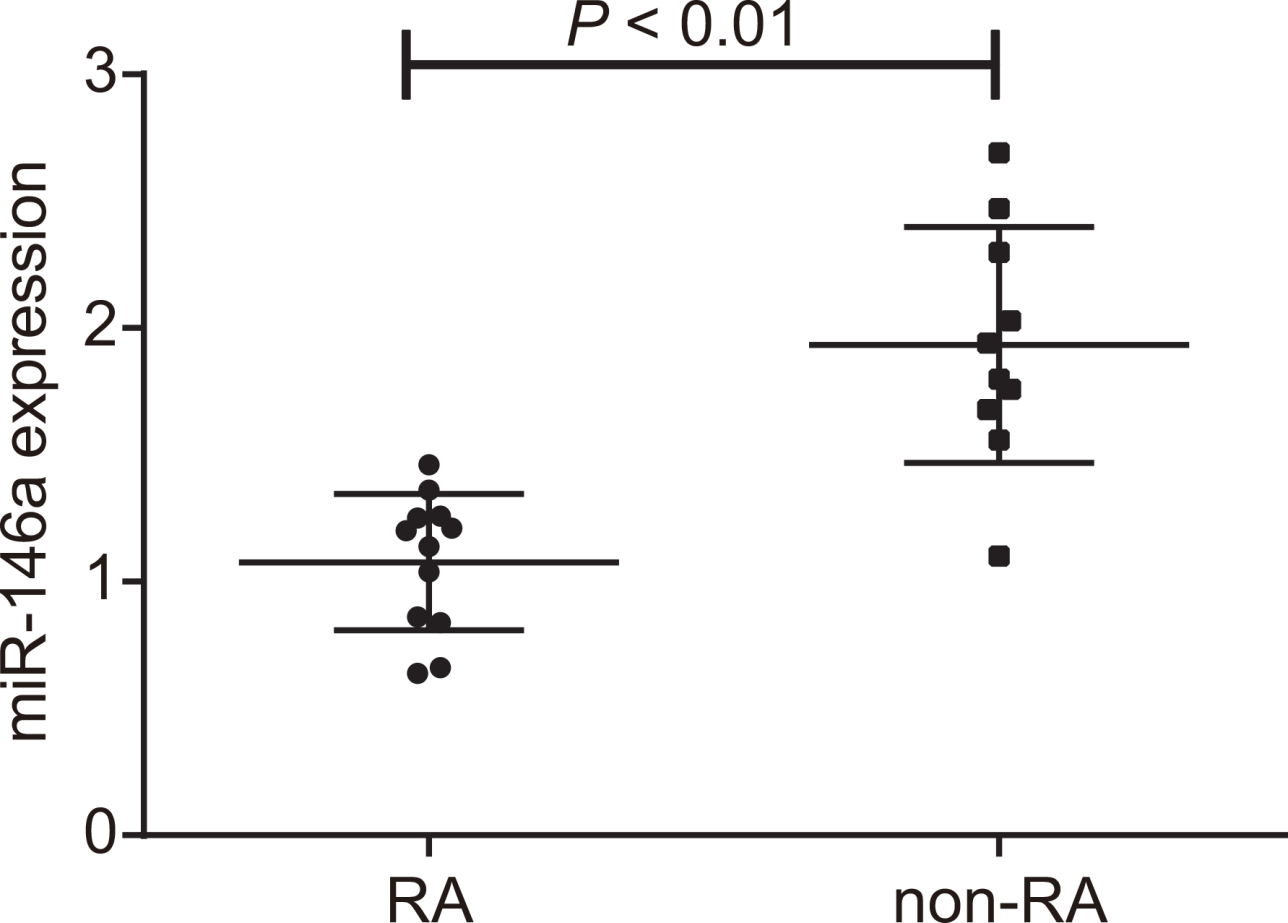
miR-146a expression in fibroblast-like synoviocytes of RA patients RT-qPCR analysis of miR-146a expression in the fibroblast-like synoviocytes from RA (*n* = 12) and non-RA (*n* = 10) patients is shown in Figure 1. Note: RA-FLS: rheumatoid arthritis fibroblast-like synoviocytes obtained from patients in First Teaching Hospital of Tianjin University of Traditional Chinese Medicine that were treated with arthroscopic synovectomies; non-RA FLS: non-rheumatoid arthritis fibroblast-like synoviocytes obtained from patients that were treated with arthroscopy for amputation due to meniscus injury to knee joint, pure meniscus injury to knee joint, anterior cruciate ligament injury, loose body, and synovial plica syndrome; ^*^denotes *P* < 0.05 in comparison to the non-RA group; miR-146a: microRNA-146a; RA: rheumatoid arthritis.

In order to investigate the correlation between miR-146a and RA, RA-FLS (*n* = 12) tissues and non-RA FLS (*n* = 10) tissues were obtained from patients. The reverse transcription quantitative polymerase chain reaction (RT-qPCR) results of miR-146a expression are shown in Figure 1. The expression of miR-146a was 1.07 ± 0.27 in RA-FLS tissues, and 1.93 ± 0.47 in non-RA FLS tissues. The expression of miR-146a was lower in RA-FLS tissues than that of non-RA FLS tissues (*P* < 0.01).

### Expression of miR-146a in RA-FLS tissues and its relationship with clinicopathological features of RA

Data was analyzed to figure out the relationship between miR-146a and clinicopathological features of RA. Comparisons between two groups were analyzed by *t-*test. Comparisons among multiple groups were analyzed by one-way analysis of variance (ANOVA). As shown in Table 1, we observed lower miR-146a expression in the RA-FLS tissues (1.07 ± 0.27) than in non-RA FLS tissues (1.93 ± 0.47; *P* < 0.05). Moreover, miR-146a expression correlated with smoking and serious and active RA disease (both *P* < 0.05). miR-146a levels were not associated with gender, age and course of disease (all *P* > 0.05).

**Table 1:** The correlation between miR-146a expression level and clinicopathological features in the RA patients

Group	Case	miR-146a	*t*	*P*
Gender			0.479	0.642
Male	3	1.14 ± 0.11		
Female	9	1.05 ± 0.31		
Age			0.556	0.591
> 60 years old	6	1.03 ± 0.33		
≦ 60 years old	6	1.12 ± 0.22		
Smoking			2.326	0.042
Yes	3	0.81 ± 0.28		
No	9	1.16 ± 0.21		
Course of disease			0.558	0.589
> 5 years	7	1.04 ± 0.32		
≦ 5years	5	1.13 ± 0.19		
Severity			9.917	0.005
Mild	2	1.41 ± 0.07		
Moderate	4	1.22 ± 0.05		
Serious	6	0.87 ± 0.22		
Types of disease activity			3.682	0.004
Active stage	6	1.88 ± 0.23		
Non-active stage	6	1.27 ± 0.12		

Note: miR-146a: microRNA-146a; Comparisons between two groups were analyzed by *t*-test. Comparisons among multiple groups were analyzed by one-way analysis of variance (ANOVA).

### MicroRNA-146a binds TLR4-3’UTR

**Figure 2:**
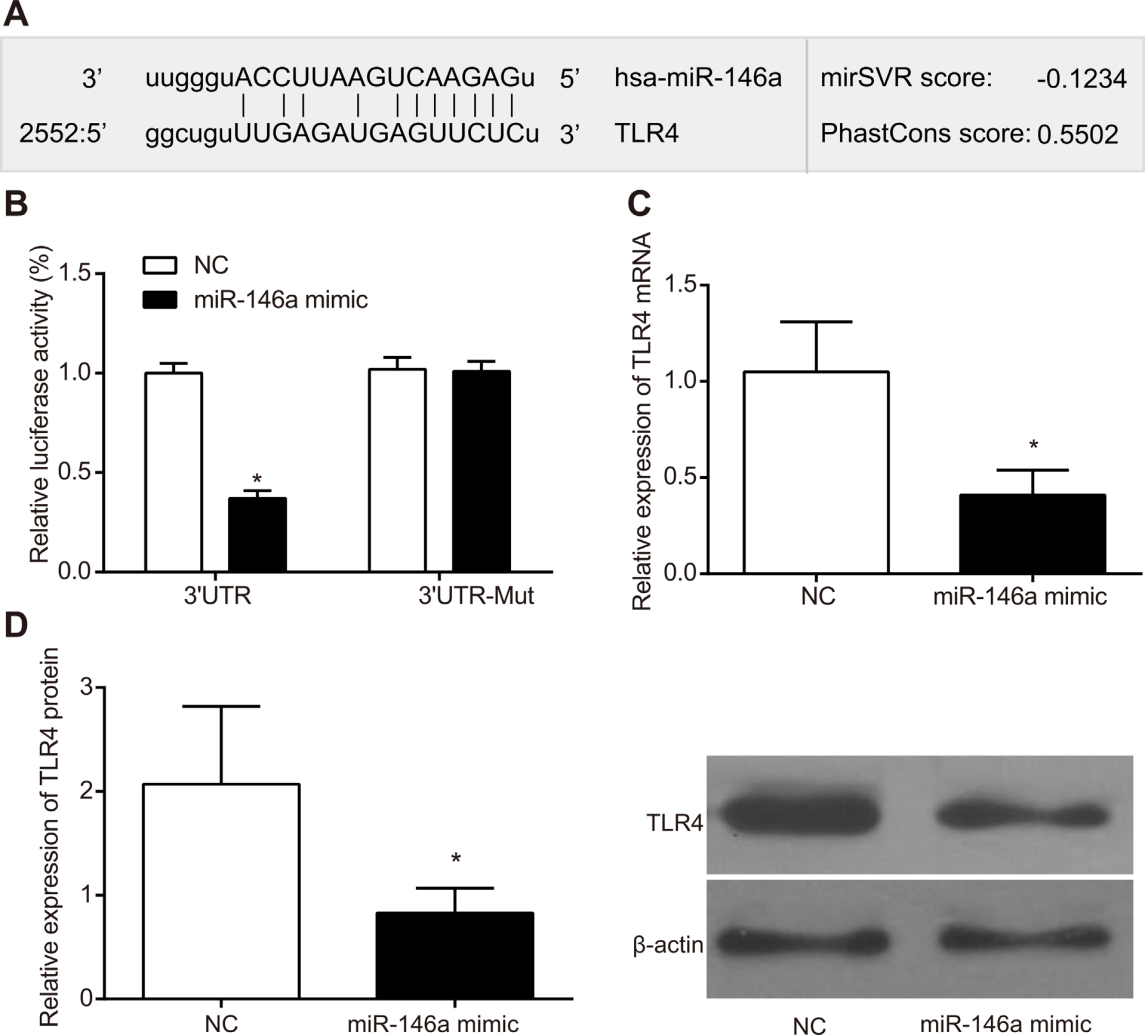
miR-146a binds to 3’UTR of *TLR4* gene (**A**) Schematic representation of Target Scan analysis demonstrating the miR-146a binding site in 3’UTR of *TLR4*. (**B**) Dual luciferase reporter assay showing luciferase activity from (i) miR-146a mimic and wild type *TLR4*-3’UTR transfected FLSs and (ii) miR-146a mimic and mutant *TLR4*-3’UTR transfected FLSs in comparison with their corresponding controls. (**C**) The mRNA expression of TLR4 in each group detected by RT-qPCR. (**D**) The protein expression of TLR4 in each group detected by Western blotting. As shown, miR-146a binds to wild type *TLR4*-3’UTR and diminishes relative firefly luciferase activity. Note: ^*^denotes *P* < 0.05 when compared with the control group.

The dual luciferase gene assay was used to confirm the target relationship between TLR4 and miR-146a. In the RA-FLS cells transfected with wild type 3’UTR of TLR4 gene, the miR-146a mimic group decreased luciferase activity compared to the negative control (NC) group (*P* < 0.05; Figure 2). In the FLS cells transfected with mutated 3’ UTR of TLR4 gene, the luciferase activities between the miR-146a mimic and the NC groups were comparable (*P* > 0.05; Figure 2). These results demonstrated that miR-146a negatively regulated TLR4 expression by binding to its 3’UTR in RA-FLS. Results of RT-qPCR and western blotting showed that the TLR4 mRNA and protein expressions were significantly lower in the miR-146a group than that of the NC group (both *P* < 0.05).

### Effect of miR-146a on expressions of TLR4 and NF-κB in RA-FLSs

**Figure 3:**
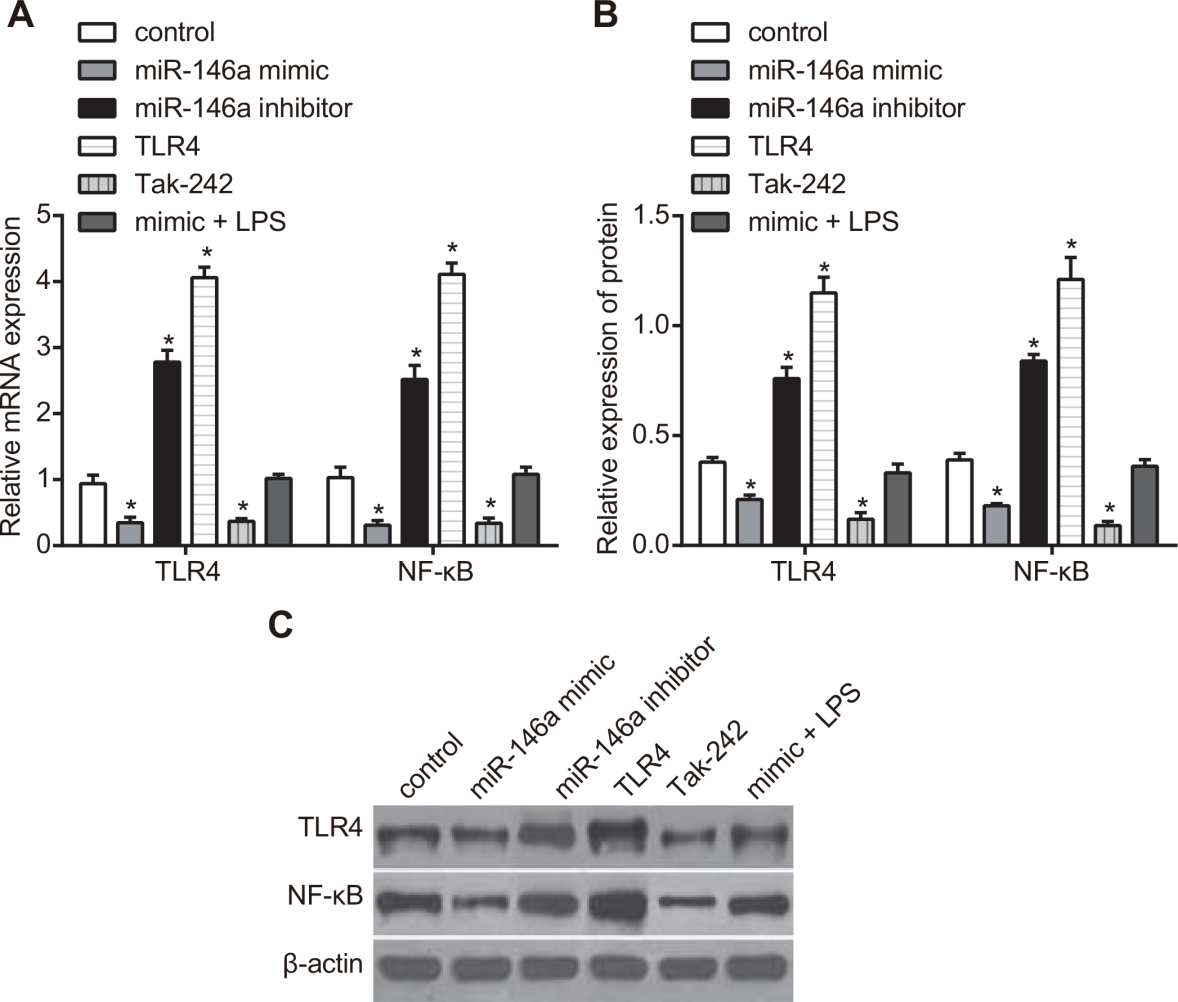
Effect of miR-146a on TLR4 and NF-κB expression in RA-FLSs (**A**) RT-qPCR analysis of TLR4 and NF-κB mRNA levels in RA-FLS cells from control, miR-146a mimic, miR-146a inhibitor, Tak-242 and mimic + LPS treatment groups. (**B**) Western blotting analysis of TLR4 and NF-κB protein levels in RA-FLS cells from control, miR-146a mimic, miR-146a inhibitor, Tak-242 and mimic + LPS treatment groups. (**C**) Representative western blots showing TLR4 and NF-κB protein levels in control, miR-146a mimic, miR-146a inhibitor, Tak-242 and mimic + LPS treatment groups. Notes: ^*^denotes *P* < 0.05 as compared with the control group. The experiment was repeated thrice with FLS cells from different groups of RA patients. Each group had four wells from different patients every time, and cell samples form 12 patients were used in the experiments.

The protein and mRNA expressions of TLR4 and NF-κB were detected by RT-qPCR and western blotting to figure out the effect of miR-146a on expressions of TLR4 and NF-κB in RA-FLSs. As shown in Figure 3, the protein and mRNA expressions of TLR4 and NF-κB decreased in the miR-146a mimic and the Tak-242 groups, while mRNA and protein expressions of TLR4 and NF-κB increased in the miR-146a inhibitor and the TLR4 groups, and the mRNA and protein expressions of TLR4 and NF-κB in the TLR4 group were significantly higher than those in the miR-146a inhibitor group (all *P* < 0.05). However, no obvious difference was found in the mimic + LPS group and the control group (*P* > 0.05).

### The levels of IL-1β, IL-6, IL-8 and IL-17 in RA-FLSs in six groups after transfection

The levels of IL-1β, IL-6, IL-8 and IL-17 were test by RT-qPCR and enzyme linked immunosorbent assay (ELISA) and collected to investigate the effect of miR-146a on them. The mRNA levels of IL-1β, IL-6, IL-8 and IL-17 reduced in the miR-146a mimic and the Tak-242 groups (all *P* < 0.05), increased in the miR-146a inhibitor and the TLR4 groups, the mRNA levels of IL-1β, IL-6, IL-8 and IL-17 were significantly higher in the TLR4 group than in the miR-146a inhibitor group (all *P* < 0.05), and no obvious differences were found between the control and the mimic + LPS groups (all *P* > 0.05; Table 2). Results of ELISA show in Table 3. Compared to the control group, the levels of IL-1β, IL-6, IL-8 and IL-17 were significantly reduced in the miR-146a mimic and the Tak-242 groups (all *P* < 0.05), while they were elevated in the miR-146a inhibitor and the TLR4 groups (all *P* < 0.05). In comparison to the control group, the levels of IL-1β, IL-6, IL-8 and IL-17 in the mimic + LPS group showed no significant difference (all *P* > 0.05). The levels of IL-1β, IL-6, IL-8 and IL-17 in the miR-146a inhibitor group were lower than in the TLR4 group (all *P* < 0.05).

**Table 2:** The mRNA levels of IL-1β, IL-6, IL-8 and IL-17 in FLS among six groups

Group	IL-1β	IL-6	IL-8	IL-17
Control	0.99 ± 0.01	0.98 ± 0.02	0.96 ± 0.04	0.97 ± 0.05
miR-146a mimic	0.38 ± 0.04^*^	0.32 ± 0.03^*^	0.36 ± 0.05^*^	0.35 ± 0.06^*^
miR-146a inhibitor	1.54 ± 0.09^*^	1.41 ± 0.08^*^	1.65 ± 0.14^*^	1.78 ± 0.17^*^
Tak-242	0.36 ± 0.05^*^	0.31 ± 0.02^*^	0.33 ± 0.04^*^	0.34 ± 0.07^*^
mimic + LPS TLR4	0.97 ± 0.03 1.86 ± 0.07^*#^	0.96 ± 0.03 1.74 ± 0.09^*#^	0.97 ± 0.02 1.98 ± 0.11^*#^	0.98 ± 0.07 2.09 ± 0.11^*#^

Note: ^*^P < 0.05 compared with the control group; ^#^*P* < 0.05 compared with the miR-146a inhibitor group; miR-146a: microRNA-146a; IL: interleukin. The experiment was repeated for three times. Each group had four wells from different patients every time, and cell samples form 12 patients were used in the experiments.

**Table 3:** The levels of proinflammatory cytokines in supernatant in FLS among each groups (ng/L)

Group	IL-1β	IL-6	IL-8	IL-17
Control	5.87 ± 0.33	6.82 ± 0.79	49.38 ± 13.57	16.28 ± 5.87
MiR-146a mimic	3.27 ± 0.23^*^	3.65 ± 0.32^*^	29.47 ± 5.76^*^	6.23 ± 1.02^*^
MiR-146a inhibitor	9.12 ± 0.56^*^	8.94 ± 0.53^*^	79.64 ± 14.52^*^	31.67 ± 8.59^*^
Tak-242	3.23 ± 0.27^*^	3.71 ± 0.34^*^	29.51 ± 5.73^*^	6.17 ± 1.07^*^
Mimic + LPS	5.85 ± 0.41	6.78 ± 0.83	50.02 ± 12.85	15.77 ± 6.16
TLR4	10.09 ± 0.11^*#^	9.96 ± 0.21^*#^	106.57 ± 7.13^*#^	56.45 ± 4.01^*#^

Note: ^*^P < 0.05 compared with the control group; ^#^*P* < 0.05 compared with the miR-146a inhibitor group; miR-146a: microRNA-146a; IL: interleukin. The experiment was repeated for three times. Each group had four wells from different patients every time, and cell samples form 12 patients were used in the experiments.

### Effect of miR-146a on nitric oxide (NO) production and inducible nitric oxide synthase (iNOS) expression in RA-FLSs

**Figure 4:**
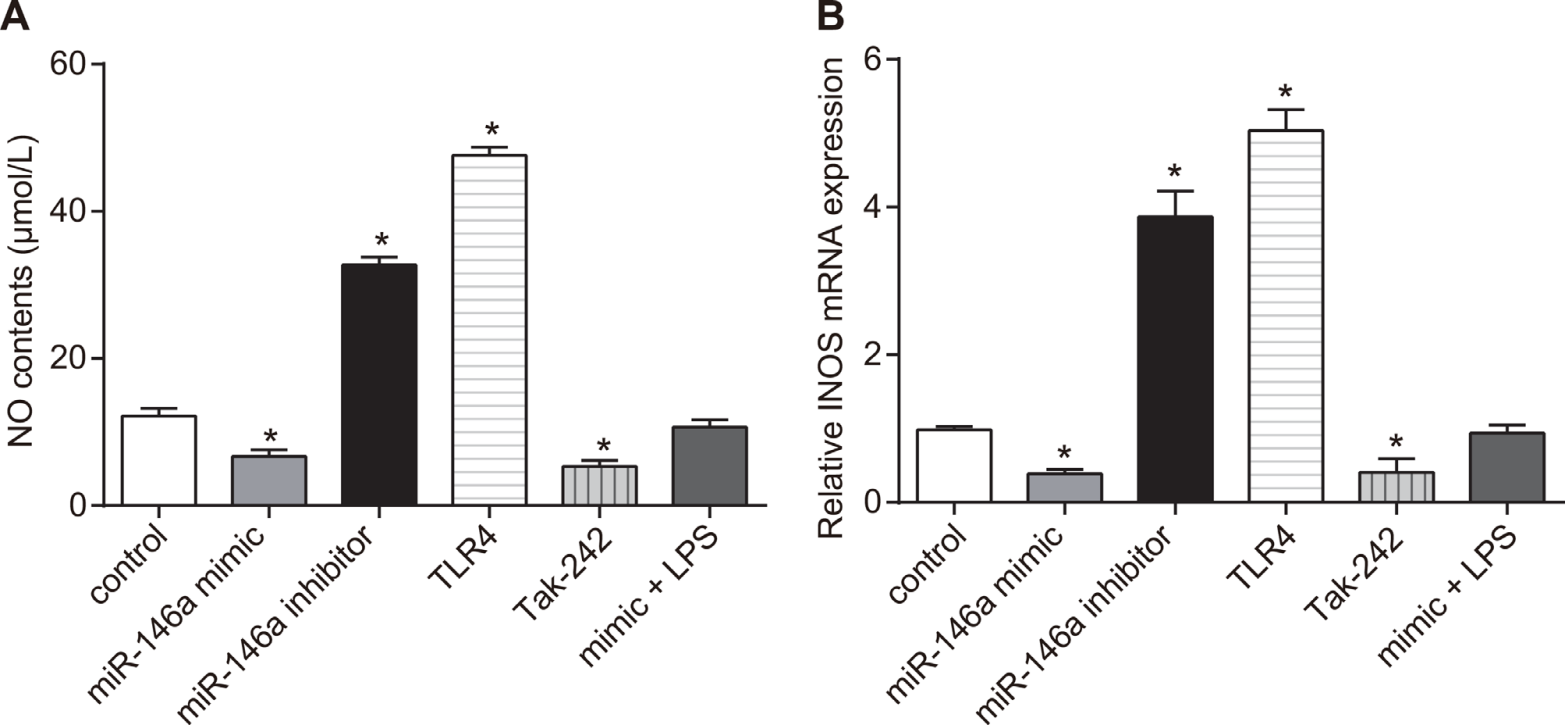
Effect of miR-146a on nitric oxide (NO) levels and inducible nitric oxide synthase (iNOS) expression in RA-FLSs (**A**) Nitric oxide levels in RA-FLS cells from control, miR-146a mimic, miR-146a inhibitor, Tak-242 and mimic + LPS treatment groups as analyzed by the Griess method. (**B**) RT-qPCR analysis of iNOS mRNA levels in RA-FLS cells from control, miR-146a mimic, miR-146a inhibitor, Tak-242 and mimic + LPS treatment. Notes: ^*^denotes *P* < 0.05 compared with the control group. RT-qPCR: reverse transcription quantitative polymerase chain reaction; NO: nitric oxide; iNOS: inducible nitric oxide synthase. The experiment was repeated thrice with FLS cells from different groups of RA patients. Each group had four wells from different patients every time, and cell samples form 12 patients were used in the experiments.

In order to figure out the role of miR-146a on the expression of NO and iNOS, the release of NO was determined by Greiss reaction and the mRNA expression of iNOS was detected via RT-qPCR. NO levels descended in the miR-146a mimic and the Tak-242 groups (*P* < 0.05), ascended in the miR-146a inhibitor and the TLR4 groups, and NO levels in the TLR4 group was higher than in the miR-146a inhibitor group (*P* < 0.05), but were similar to control in the mimic + LPS group (*P* > 0.05; Figure 4A). The iNOS mRNA levels decreased in the miR-146a mimic and the Tak-242 groups (*P* < 0.05), increased in the miR-146a inhibitor and TLR4 groups, and iNOS levels in the TLR4 group was higher than in the miR-146a inhibitor group (both *P* < 0.05), but were similar to control in the mimic + LPS group (*P* > 0.05; Figure 4B).

### Effect of miR-146a on expression of COX-2, PGE2, MMP-3 and Seprase in RA-FLSs

**Figure 5:**
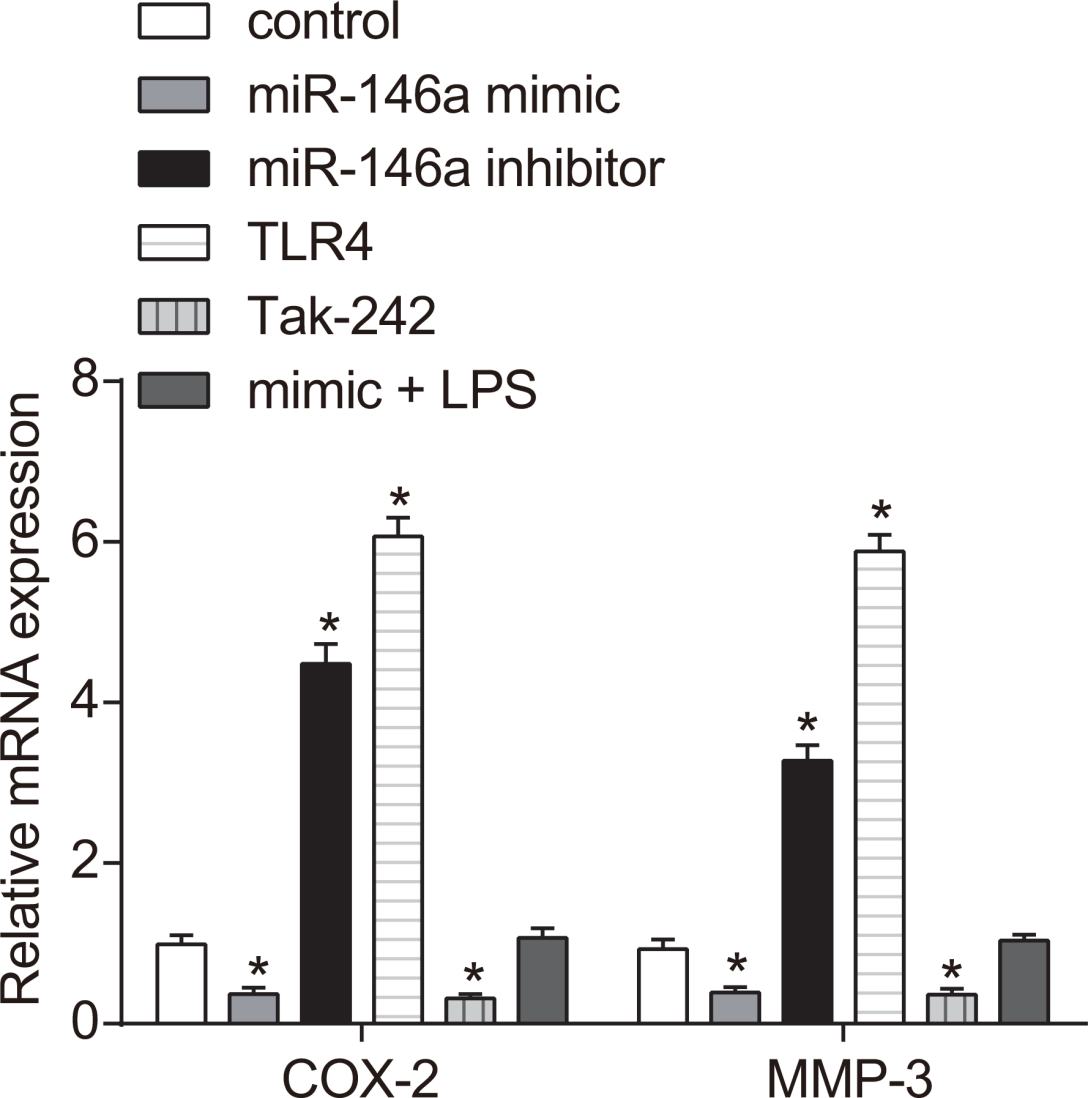
Effect of miR-146a on COX-2, PGE2, MMP-3 and Seprase expression in RA-FLSs RT-qPCR and western blotting analysis of COX-2, PGE2, MMP-3 and Seprase mRNA and protein levels in RA-FLS cells from control, miR-146a mimic, miR-146a inhibitor, Tak-242 and mimic + LPS treatment groups. Notes: ^*^denotes *P* < 0.05 compared with the control group. COX-2: cyclooxygenase-2; MMP3: matrix metalloproteinase-3.

Effect of miR-146a on expressions of COX-2, PGE2, MMP-3 and Seprase in RA-FLSs could be investigated. The mRNA expressions of COX-2 and MMP-3 were detected by RT-qPCR and ELISA was used to measure the expressions of Seprase and MMP-3. COX-2 and MMP-3 mRNA levels decreased in the miR-146a mimic and the Tak-242 groups, but increased in the TLR4 and miR-146a inhibitor groups and COX-2 and MMP-3 expressions in the TLR4 group were higher than in the miR-146a inhibitor group (all *P* < 0.05) and were similar to control in the mimic + LPS groups (*P* > 0.05; Figure 5). ELISA results demonstrated that PGE2, MMP-3 and Seprase protein expressions were decreased in the miR-146a mimic and the Tak-242 groups, increased in the miR-146a inhibitor and the TLR4 groups (all *P* < 0.05), the protein expressions of PGE2, MMP-3 and Seprase were higher in the TLR4 group than that of the miR-146a inhibitor group, and similar to control in the mimic + LPS groups (*P* > 0.05; Table 4).

**Table 4:** The levels of PGE2, MMP-3, and Seprase in FLS among six groups after transfection (ng/L)

Group	PGE2	MMP-3	Seprase
Control	9.76 ± 2.15	185.84 ± 15.27	0.65 ± 0.08
MiR-146a mimic	3.21 ± 1.26^*^	76.41 ± 16.32^*^	0.31 ± 0.06^*^
MiR-146a inhibitor	31.72 ± 1.53^*^	391.75 ± 21.38^*^	1.29 ± 0.07^*^
Tak-242	3.28 ± 1.19^*^	77.68 ± 15.94^*^	0.33 ± 0.05^*^
Mimic + LPS	10.08 ± 2.38	187.62 ± 16.46	0.72 ± 0.13
TLR4	47.45 ± 1.01^*#^	54.11 ± 14.53^*#^	7.49 ± 0.09^*#^

Note: ^*^P < 0.05 compared with the control group; ^#^*P* < 0.05 compared with the miR-146a inhibitor group; miR-146a: microRNA-146a; MMP-3: matrix metalloproteinase-3; PGE-2: prostaglandin E2. The experiment was repeated for three times. Each group had four wells from different patients every time, and cell samples form 12 patients were used in the experiments.

### Effects of miR-146a on RA-FLS morphology

**Figure 6:**
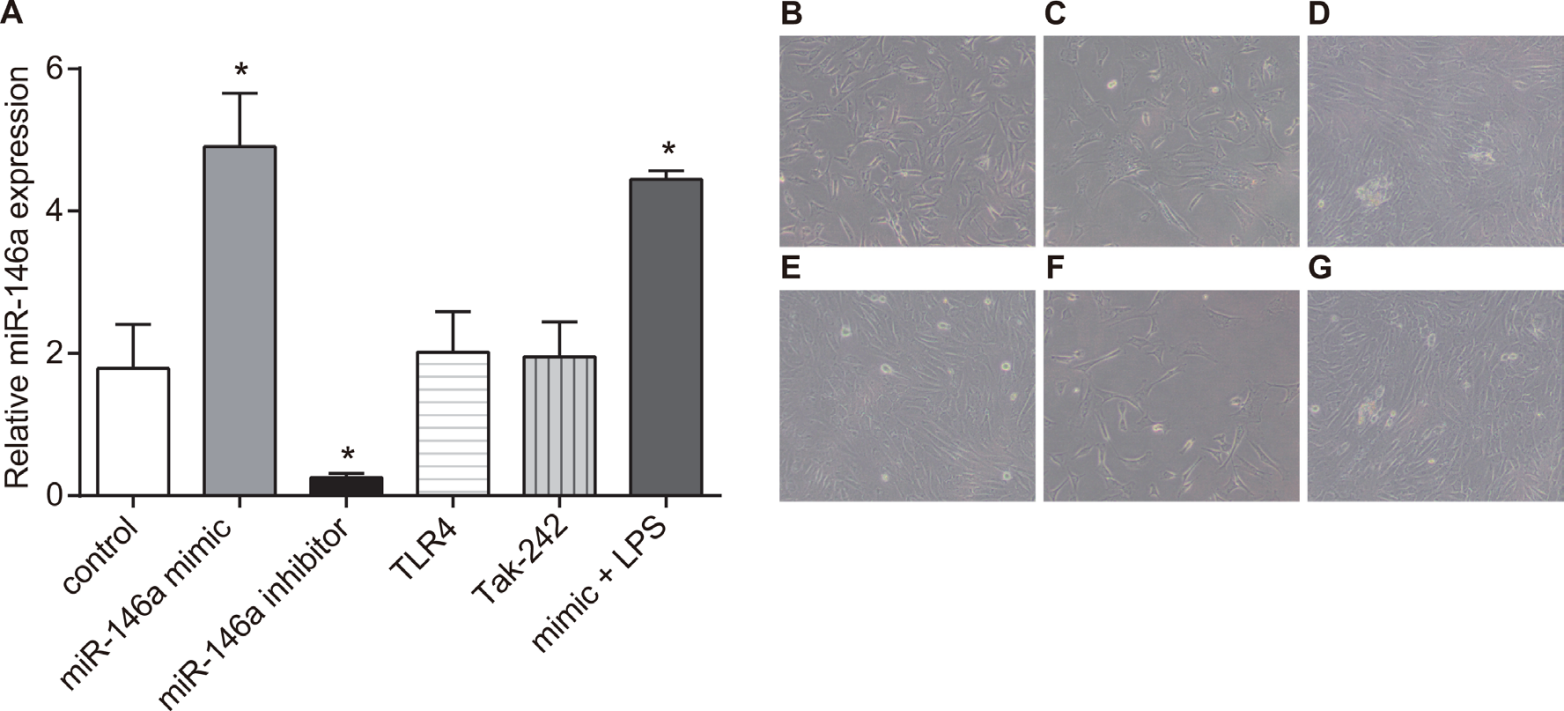
Effects of miR-146a on RA-FLSs morphology (× 200) Twelve different synovial cells were selected from the original cells. The cells were randomly divided into 3 groups (*n* = 4) that was repeated three times, each time each experiment group from four different patients with complex wells, a total of 12 patients from the cell samples were used for the experiment. (**A**) miR-146a expression in six group. Representative images show morphology of RA-FLS cells in (**B**) control; (**C**) miR-146a mimic; (**D**); (**E**) Tak-242; (**F**) mimic + LPS; (**G**) TLR4 groups; ^*^denotes *P* < 0.05 as compared with the control group.

In order to investigate effects of miR-146a on RA-FLSs morphology, the expression of miR-146a was detected by RT-qPCR, and the morphology of miR-146a was observed by an inverted microscope. Results of RT-qPCR showed that the control and miR-146a mimic + LPS treated RA-FLS cells showed dense cell growth and similar cell morphology. In the miR-146a mimic and Tak-242 treatments, the RA-FLSs were smaller with high refractive index, decreased cell density and apoptotic. The miR-146a inhibitor and TLR4 treated RA-FLSs showed increased cell density with closely arranged cells (Figure 6).

### miR-146a overexpression reduces FLS cell proliferation

**Figure 7:**
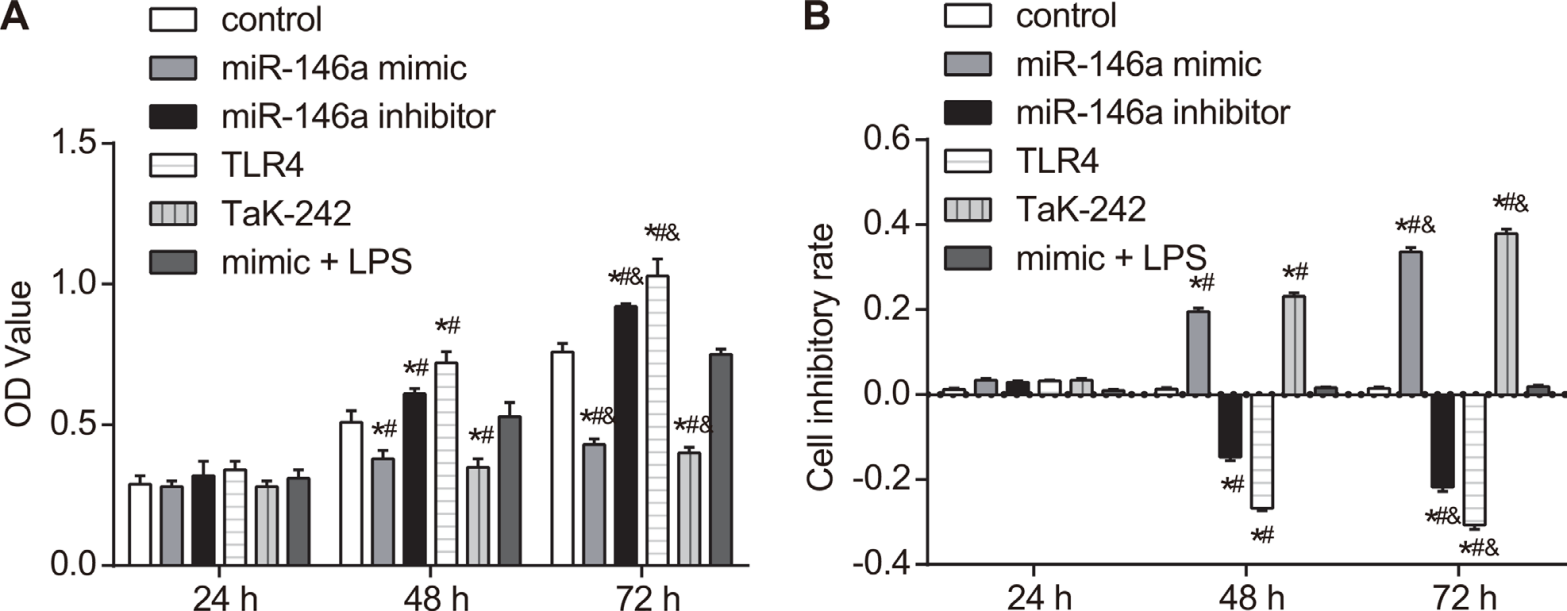
Effect of miR-146a on RA-FLS cell proliferation (**A**) CCK-8 assay shows proliferation of RA-FLS cells from control, miR-146a mimic, miR-146a inhibitor, Tak-242 and mimic + LPS treatment groups at 24, 48 and 72 h. (**B**) Cell inhibitory rate in control, miR-146a mimic, miR-146a inhibitor, Tak-242 and mimic + LPS treatment groups at 24, 48 and 72 h. Note: ^*^denotes *P* < 0.05 as compared with the control group; ^#^denotes *P* < 0.05 as compared with the 24 h time point; ^&^denotes *P* < 0.05 as compared with the 48 h time point; CCK-8: cell counting kit-8; OD: optical density. The experiment was repeated thrice with cells from different RA patients. Each group had four wells from different patients every time, and cell samples form 12 patients were used in the experiments.

Cell counting kit (CCK-8) assay was used to detect the effect of miR-146a on FLS cell proliferation. CCK-8 assay showed that each group had similar rates of proliferation at 24 h (all *P* > 0.05; Figure 7). However, cell proliferation increased in the miR-146a inhibitor and the TLR4 groups (both *P* < 0.05) and decreased in the miR-146a mimic and the Tak-242 groups (both *P* < 0.05) than in the control group at 48 and 72 h (*P* > 0.05). As the time of culture increased, the proliferation increased in all groups (all *P* < 0.05), and the inhibitory rate decreased in the miR-146a inhibitor and the TLR4 groups (both *P* < 0.05), while increased in the miR-146a mimic and the Tak-242 groups (both *P* < 0.05). No significant difference was found in the mimic + LPS group and the control group (Figure 7).

### Effect of miR-146a on RA-FLSs apoptosis

**Figure 8:**
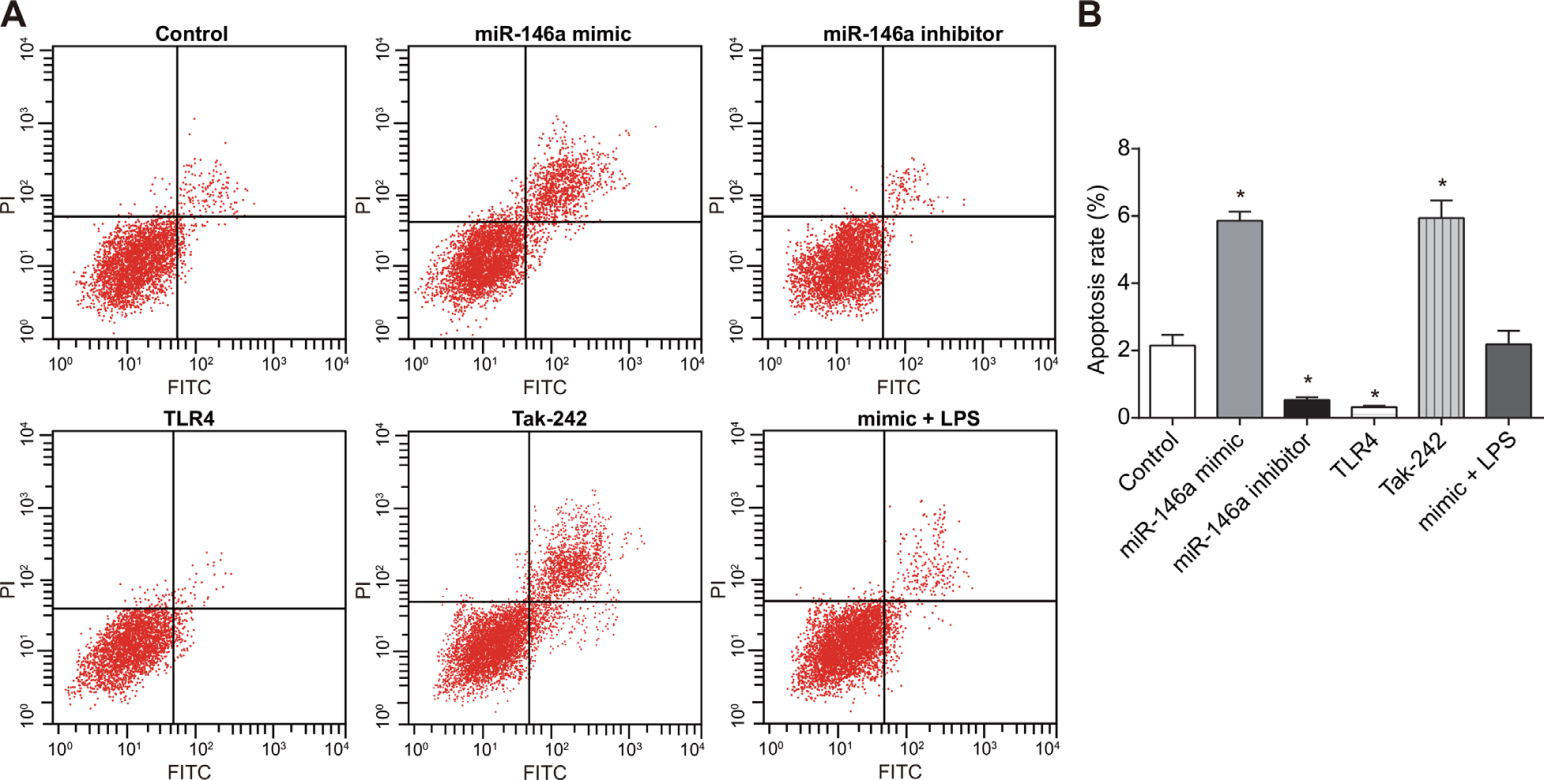
Effect of miR-146a on RA-FLS cell apoptosis (**A**) FACS plots showing AnnexinV-FITC versus propidium iodide staining in RA-FLSs from control, miR-146a mimic, miR-146a inhibitor, Tak-242 and mimic + LPS treatment groups. (**B**) Apoptotic rate (percent AnnexinV^+^ PI^+^ cells) of FLSs in control, miR-146a mimic, miR-146a inhibitor, Tak-242 and mimic + LPS treatment groups. Note: ^*^denotes *P* < 0.05 compared with the control group. The experiment was repeated thrice with FLS cells from different groups of RA patients. Each group had four wells from different patients every time, and cell samples form 12 patients were used in the experiments.

In order to investigate effect of miR-146a on RA-FLSs apoptosis, Annexin V/PI double staining was used to detect the apoptotic rate. As shown in Figure 8, the apoptotic rate (percent Annexin V^+^ PI^+^ apoptotic cells) increased in the miR-146a mimic sand the Tak-242 groups, but decreased in the miR-146a inhibitor and thr TLR4 groups, and apoptotic rate in the TLR4 group was lower than in the miR-146a inhibitor group (*P* < 0.05) and was similar to control in the mimic + LPS group (*P* > 0.05). However, there was no difference in the apoptotic cells between the miR-146a mimic and the Tak-242 groups (*P* > 0.05).

### MiR-146a overexpression inhibits inflammatory response of FLSs in RA model rats

**Figure 9:**
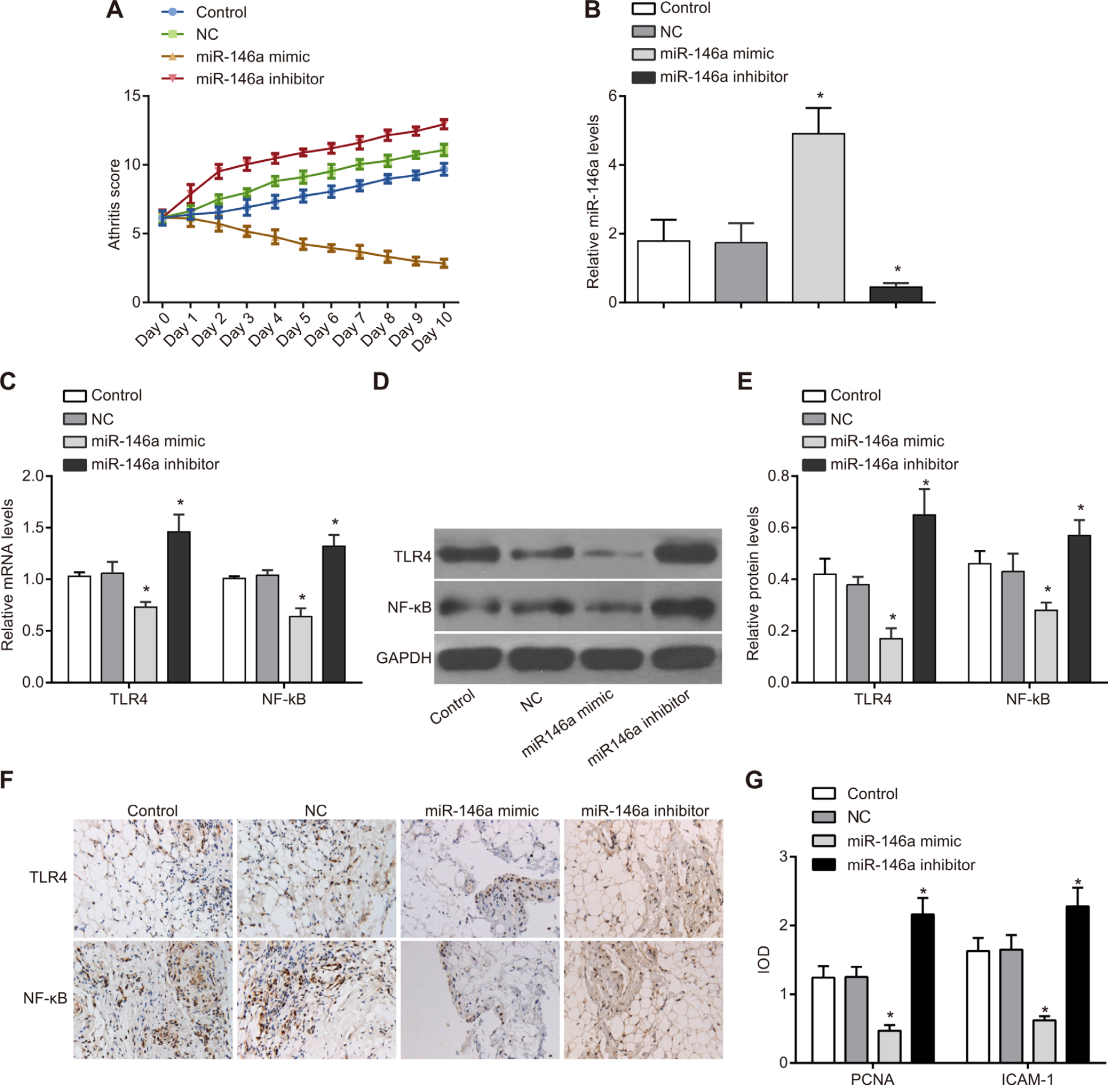
Effect of miR-146a on inflammatory response in rat RA model (**A**) Arthritis score of RA model rats transplanted with control, negative control, miR-146a mimic and miR-146a inhibitor transfected rat RA-FLSs. (**B**) miR-146a expression in tissues of RA model rats from control, negative control, miR-146a mimic and miR-146a inhibitor transfected rat RA-FLSs. (**C**) TLR4 and NF-κB mRNA levels in tissues of RA model rats from control, negative control, miR-146a mimic and miR-146a inhibitor transfected rat RA-FLSs. (**D**) TLR4 and NF-κB protein levels in tissues of RA model rats from control, negative control, miR-146a mimic and miR-146a inhibitor transfected rat RA-FLSs. (**E**) Statistics of TLR4 and NF-κB mRNA levels in tissues of RA model rats from control, negative control, miR-146a mimic and miR-146a inhibitor transfected rat RA-FLSs. (**F**) Immunohistochemistry results of PCNA and ICAM-1 in FLS tissues of each group (× 200). (**G**) The expression levels of PCNA and ICAM-1 in FLS tissues (expressed as IOD values). Note: ^*^denotes *P* < 0.05 compared with the control group.

In order to figure out the effect of miR-146a on the symptoms of RA rats, the RA rat model was established. After the group treatment was finished, and the score of the inflammation was counted. In the rat RA model, the arthritis score was lower in the miR-146a mimic group than in the control and NC groups suggesting that miR-146a overexpression decreased inflammation. Moreover, lower TLR4 and NF-κB mRNA and protein levels were observed in the miR-146a mimic group than in the control and NC groups (*P* < 0.05) (Figure 9). Results of immunohistochemistry showed that compared with the control and the NC groups, the expressions of proliferating cell nuclear antigen (PCNA) and intercellular adhesion molecular-1 (ICAM-1) in RA-FLSs were significantly declined in the miR-146a mimic group, but elevated in the miR-146a inhibitor groups (both *P* < 0.05). These data suggested that miR-146a suppressed the inflammatory response and down-regulated the expression of proliferation-related molecules in rat RA-FLSs by inhibiting the TLR4/NF-κB signaling pathway.

## DISCUSSION

In this study, we investigated the role of miR-146a and TLR4/NF-κB signaling pathway in the proliferation and pro-inflammatory cytokine production of RA-FLSs. RA-FLSs present increased expression of both proinflammatory genes and matrix-destructive enzymes, together with numerous alterations in cell signaling [18]. A previous study reported that miR-146a could control TLR and cytokine signaling through targeting the TNF receptor–associated factor 6 and IL-1 receptor-associated kinase [17]. Similarly, our results demonstrated that miR-146a overexpression inhibits RA-FLS cell proliferation and pro-inflammatory cytokine production by inhibiting the TLR4/NF-κB signaling pathway. Li *et al.* showed that miR-192 was downregulated in RA synovial tissues [19]. Nakasa *et al.* reported that miR-146a was induced by LPS and proinflammatory mediators [20]. MiR-146a targets interleukin-1 receptor-associated kinase (IRAK1) and modulates the function of toll-like receptors and related cytokines [21]. Pauley *et al.* showed that miR-146a expression controlled the release of some pro-inflammatory cytokines and therefore regulated the extent of stimulation [22]. TLRs recognize many extracellular molecules associated with pathogens or tissue damage and activate transcription factors that promote expression of inflammatory factors [23]. When TLR4 is bound by its ligand, it activates NF-κB through myeloid differentiation factor-88 (MyD88) dependent and independent pathways [24]. The NF-κB family of transcription factors translocate from the cytoplasm into the nucleus upon activation to induce cytokine expression [25]. These cytokines promote the intracellular expression of iNOS mRNA and the release of NO, which promotes inflammation [26]. Therefore, lower miR-146a expression promotes the release of proinflammatory cytokines, thus exacerbating RA pathology.

We also found that lower miR-146a expression was associated with increased FLS proliferation and increased levels of the fibroblast growth factors, COX-2, PGE2, MMP-3 and Seprase. When the TLR4/NF-κB pathway is activated, NF-κB enters the nucleus and upregulates the expression of transforming growth factor-β or TGF-β [27]. In addition, autocrine and paracrine secretion of NF-κB promotes the aggregation of extracellular matrix and induces the synthesis of related matrix metalloproteinases, including MMP-3, COX-2, PGE2 and Seprase [27]. MMP-3 belongs to a group of proteolytic enzymes that degrade extracellular matrix proteins causing ligament or cartilage damage [28]. Increased MMP-3 activity leads to fibrosis [29]. COX2 and PGE2 are both activated by inflammatory responses and promote the formation of fibroblasts [30]. Seprase is a member of the serine protease family with dipeptidyl peptidase and collagenase activities, which is often highly expressed in human malignant tumors [31]. Seprase increases protein cleavage in cells and accelerates fibrosis by activating human fibroblasts [32]. Therefore, activation of the TLR4/NF-κB pathway both directly and indirectly increases the expression of growth factors that promote tissue fibrosis. Our study demonstrates that miR-146a suppresses FLS proliferation by downregulating the activation of the TLR4/NF-κB signaling pathway.

In summary, our study shows that miR-146a reduces FLS cell proliferation and pro-inflammatory cytokine production by inhibiting TLR4/NF-κB signaling, thereby suppressing RA. Therefore, miR-146a and TLR4/NF-κB signaling are potential therapeutic targets for RA. Here, we focused on association between miR-146a and the TLR/NK-κB signaling pathway specifically; future studies should examine the mechanisms by which miR-146a is regulated and identify its target molecules. Moreover, the signaling pathway that could regulate miR-146a in the FLSs was not clear, which has not been reported. We have great interests in this research, and we would study it in the future. In addition, clinical studies of RA patients are needed to expand upon these results.

## MATERIALS AND METHODS

### Study subjects

FLSs were obtained from RA patients (*n* = 12; 3 males and 9 females; age: 39 ∼ 74 year; mean age: 58.42 year) that underwent arthroscopic synovectomies in the Department of Orthopedics at People’s Hospital of Tianjin. All patients met the RA classification criteria according to American College of Rheumatology [33] and had no previous history of complicated diseases of the heart, brain, liver or kidney, traumatic osteoarthritis, purulent osteoarthritis, or primary knee osteoarthritis. Non-RA FLSs were obtained from patients (*n* = 10; 1 male and 9 females; age: 38∼71 year; mean age: 55.90 year) that were treated with arthroscopy for amputation due to meniscus injury to knee joint, pure meniscus injury to knee joint, anterior cruciate ligament injury, loose body, and synovial plica syndrome. There were no significant differences in age and gender between two groups (both *P* > 0.05). After surgery, all tissue samples were transferred quickly to Dulbecco’s modified Eagle’s medium (DMEM, Gibco Company Grand Island, NY, USA) containing 100 U/mL penicillin, 100 µg/mL streptomycin, and 10% fetal bovine serum (FBS) (Gibco Company Grand Island, NY, USA). The experiments were approved by the Ethics Committee of First Teaching Hospital of Tianjin University of Traditional Chinese Medicine, and all patients provided informed consent.

### Dual luciferase reporter gene assay

We analyzed the target genes of miR-146a at the microRNA.org database and identified TLR4 was one of them. Then, FLSs were transfected with miR-146a mimic and miR-146a NC in combination with TLR4-3’UTR plasmid vector and mutant TLR4-3’UTR vector. Dual luciferase reporter gene assay was performed according to the manufacturer’s instructions (Promega Corp., Madison, Wisconsin, USA). The FLSs were then washed twice with 500 µl PBS followed by incubation at room temperature for 15 min with 100 µl 1X passive lysis buffer (PLB). Then, cell lysate was transferred into new chemiluminescence tube with 100 µl LARII followed by 20 µl PLB. Then, after adding 100 µl 1X Stop & G1o solution, we analyzed firefly and renilla luciferase activities. Relative luciferase activity refers to the ratio of firefly luciferase activity to renilla luciferase activity. The expressions of TLR4 in the miR-146a mimic and the NC groups were detected by RT-qPCR and Western blotting.

### RA-FLS cell grouping and transfection

The miR-146a mimic (5′-UGAGAACUGAAU UCCAUGGGUU-3′), inhibitor (5′-AACCCAUGGAAUUC AGUUCUCA-3′) and TLR4 overexpressing vector were purchased from Shanghai Zimmer Pharmaceutical Company (Shanghai, China). RA-FLS cells were divided into 6 experimental groups: (1) control (no transfection); (2) miR-146a mimic (transfected with miR-146a mimic); (3) miR-146a inhibitor (transfected with miR-146a inhibitor); (4) Tak-242 (treated with TLR4/NF-κB signaling pathway inhibitor); and (5) miR-146a mimic + LPS (transfected with miR-146a mimic and treated with TLR4/NF-κB signaling pathway activator) and (6) TLR4 (treated with TLR4 overexpressing vector). The cells were transfected with lipofectamine 3000 (Invitrogen Inc., Carlsbad, CA, USA) according to the manufacturer’s instructions. A day before the transient transfection, cells were harvested by trysinization and seeded into six-well plates until they reached 90% to 95% confluence. The miR-146a mimic and inhibitor were diluted in 50 µl serum-free medium, whereas 1 µl lipofectamine 3000 reagent was diluted in 50 µl serum-free medium. The miR-146a mimic and inhibitor were then mixed with 1X lipofectamine 3000 reagent for 5 min and incubated at room temperature for 20 min. After changing the cell culture medium, the mimic or inhibitor mixtures were added into the cells and gently shaken and incubated at 37°C and 5% CO_2_ for 4 h. Then, fresh medium was added and after 24 h incubation, transfection efficiency of each group was checked under an inverted fluorescence microscope. Changes in cellular morphology were documented after 72 h.

### RT-qPCR

Total RNA was extracted from cells or tissues using the RNA extraction kit (Qiagen, Valencia, USA) according to the manufacturer’s instructions. The quantity of RNA was determined from the 260/280 OD ratio and the extracted RNA samples were stored at -80^o^C. RNA was reverse transcribed to cDNA with the RNA reverse transcription kit (Qiagen, Valencia, USA). The primers for the qPCR were designed with the Primer 5.0 software based on gene sequences from the Genbank database (Table 5). All primers were designed and synthesized by the Shanghai Biotech Engineering Company (Shanghai, China). The qPCR reaction (20 µl) consisted of 10 µl SYBR Premix ExTaq, 0.8 µl each of forward and reverse primers, 0.4 µl ROX reference dye II, 2 µl cDNA template and 6 µl distilled water. The qPCR reaction conditions were 40 cycles of 95°C for 30 s, 95°C for 5 s, and 60°C for 30 s. The PCR results were verified by the melting curve. Relative expression was calculated by the cycle threshold (Ct) values and the 2^-△△Ct^ method using β-actin as an internal control [34].

**Table 5:** The primer sequences for RT-qPCR

Gene	Upstream primer (5′∼3′)	Downstream primer (5′∼3′)
miR-146a (human)	TATTGGGCAAACAATCAGCA	GCCTGAGACTCTGCCTTCTG
miR-146a (rat)	CGCTACTCGTACCGTGAGTAA	GTGCAGGGTCCGAGGT
TLR4 (human)	ACTTGGACCTTTCCAGCAAC	TTTAAATGCACCTGGTTGGA
TLR4 (rat)	GGACTCTGCCCTGCCACCATTTA	CTTGTGCCCTGTGAGGTCGTTGA
NF-κB (human)	TCAAGATCTGCCGACTGAAC	CCTCTTTCTGCACCTTGTCA
NF-κB (rat)	GTGCAGAAAGAAGACATTGAGGTG	AGGCTAGGGTCAGCGTATGG
IL-1β (human)	GGATATGGAGCAACAAGTGG	ATGTACCAGTTGGGGAACTG
IL-6 (human)	ATGAGCTCCTTCTCCACAAGCGC	GAAGAGCCCTCAGGCTGGACTG
IL-8 (human)	CATACTCCAAACCTTTCCACCCC	TCAGCCCTCTTCAAAAACTTCTCCA
IL-17 (human)	GGGCCTGGCTTCTGTCTGA	AAGTTCGTTCTGCCCCATCA
MMP-3 (human)	GGGTGAGGACACCAGCATGA	CAGAGTCGGAGTCCAGCTTC
iNOS (human)	TCCAGGAGGACATGCAGCAC	CGCCCTTCCGCAGTTCT
COX-2 (human)	ATCTACCCTCCTCAAGTCCC	TACCAGAAGGGCAGGATACAG
β-actin (human)	TGGCACCCAGCACAATGAA	CTAAGTCATAGTCCGCCTAGAAGCA
β-actin (rat)	GAGAGGGAAATCGTGCGTGAC	CATCTGCTGGAAGGTGGACA

Notes: RT-qPCR: reverse transcription quantitative polymerase chain reaction; miR-146a: microRNA-146a TLR4: toll-like receptor-4; NF-κB: nuclear factor kappa-light-chain-enhancer of activated B cells; IL: interleukin; MMP: matrix metalloproteinase-3; iNOS: inducible nitric oxide synthase; COX-2: cyclooxygenase-2.

### Western blotting

Protein lysates were prepared from different experimental groups of cells and tissues and quantified with the bicinchoninic acid (BCA) kit (Wuhan Boster Biological Technology Co., Ltd. Wuhan, China) according to the manufacturer’s instructions. Then, equal amounts of protein (30 µg) were separated by 10% polyacrylamide gel electrophoresis (PAGE) (Wuhan Boster Biological Technology Co., Ltd. Wuhan, China) at 100V for 2 h and transferred onto PVDF membranes at 100V for 1 h. Then, after blocking the membrane with 5% skimmed milk in 1X TBST buffer, the blots were incubated overnight at 4°C with anti-TLR4 (ab30667) and anti-NF-κB (ab31432) antibodies (both from Abcam Inc., Cambridge, MA, USA; 1:1000 dilution) as well as anti- β-actin antibody (Becton, Dickinson and Company, NJ, USA; 1:3000) as internal control. Then, after washes with 1X TBST, the blots were incubated at room temperature with HRP-conjugated secondary antibodies for 2 h. After washing, the blots were developed with ECL chemiluminescence reagent and imaged with a Gel Doc EZ Imager (Bio-Rad Laboratories, Inc. CA, USA). The grey values of the protein bands were quantified with the Image J software.

### ELISA

The levels of interleukin (IL)-1β, IL-6, IL-8, IL-17, Seprase, MMP-3 and prostaglandin E2 (PGE2) in cell culture supernatants were measured by ELISA (R&D company, USA) according to the manufacturer’s instructions. The standard and experimental samples were incubated with freshly prepared 1: 99 diluted biotinylated antibodies against IL-1β, IL-6 IL-8, IL-17, Seprase, MMP-3 and PGE2 in 96 well ELISA plates at room temperature for 2 h. Then, the liquid was aspirated out and 400 µl detergent was added to each well. After washing thrice, the residual liquid was dried with absorbent paper. Then, 200 µl chromogenic substrate was added to each well, and incubation for 30 minutes in dark followed by 50 µl stopping solution. The absorbance was measured at 450 nm as well as 570 nm for the reference wavelength internal control. The concentrations of the interleukins in the supernatants of samples were determined from the corresponding standard curves multiplied by the dilution factor.

### Griess reaction

To determine nitric oxide release, we generated a standard curve with 100, 50, 25, 12.5, 6.25, and 3.125 mM NaNO_2_ solutions. The absorbance was measured at 540 nm after incubating at room temperature for 10 minutes. The amount of nitric oxide from different experimental samples was calculated based on the standard curve.

### Isolation and culture of FLSs

Fatty tissues were separated out from the RA-FLSs, which were then cut into 1 mm^3^ pieces aseptically and washed with D-Hanks solution. The tissue pieces were then incubated with 2 times volume of 1 mg/ml collagenase I (Gibco Company, Grand Island, NY, USA) for 2 h at 37°C. Then, the cell suspension was filtered with stainless steel sieves and nylon nets followed by filtration with nylon nets (70 µm pore diameter) to obtain FLS cell suspension. After centrifugation and washing with phosphate-buffered saline (PBS), the FLSs were cultured in DMEM medium containing 10% FBS until they reached 80% ∼ 90% confluence. Then, the cells were trypsinized with 0.25% EDTA-trypsin and seeded at a 1 to 3 ratio for subsequent passages. Cells from 3–5 passages, growing in the logarithmic phase were used in the experiments. The cell morphology and growth was constantly observed under an inverted microscope.

### Cell counting kit-8 (CCK-8) assay

Cell proliferation was analyzed with a CCK-8 kit (Dojindo Laboratories, Kumamoto, Japan). Cells from 3–5 passages, trypsinized with 0.25% EDTA-trypsin and grown in the logarithmic phase were used in the experiments. The FLSs (1 × 10^4^ cells/100 µl) were seeded and grown in 96-well plates and CCK-8 (10 µl) solution was added at 24, 48, and 72 h and incubated at 4°C for 1–4 h. The edge wells were filled with sterile PBS, and cultured in the incubator to make the cells adhered to the wall. When the cells reached 80% confluence, they were assigned into 6 parallel wells according to the experimental groups. The OD was measured with a microplate reader (Varioskan Flash, Thermo, USA) at the wavelength of 450 nm. The experiment was repeated 3 times, and the average OD value was obtained. The cell inhibitory rate was calculated as 1 - (OD value of experimental group/OD value of control group) × 100%.

### Flow cytometry

RA-FLS cells (1 × 10^6^ per sample) from different experimental groups were stained in 500 µl binding buffer with 5 µl PI and 5 µl AnnexinV-FITC (Nanjing KeyGen Biotech Co. Ltd., Nanjing, China) for 10–15 mins. Then, the samples were analyzed by flow cytometry in a FACS Calibur (BD Biosciences). The percent apoptotic (AnnexinV^+^ PI^+^) cells were analyzed in the different experimental groups.

### RA rat models and experimental grouping

Twenty-five Wistar Furth adult male rats (8∼12 week old and 250 ± 20 g) were purchased from Beijing Vital River Laboratory Animal Technology Co., Ltd. (Beijing, China). All the rats were housed under controlled conditions of temperature (23 ± 2°C), relative humidity (55%∼65%), illuminating for 12 h and avoiding light for 12 h, and ad libitum access to food and water. The beddings in the cages were changed every day and the cages were cleaned and disinfected. Rats were treated with adaptive feeding for 1 week before the experiment, and were then numbered. According to the program of Chondrex (WA, USA), from which bovine type II collagen was purchased, incomplete Freund’s adjuvant (IFA) was mix with collagen and emulsified. The mixture was intradermally injected into the root of tails, and it was injected again for intensifying after 7 days. In the 3rd week, 18 rats suffered arthritis of the hind paws, and 2 rats suffered arthritis of the front paws and hind paws. The arthritis symptoms in joint were stable and sustained. Then, 5 rats were randomly selected and anesthetized with intraperitoneal injection of 20% urethane (10 mg/kg), fixed on the stereotaxic instrument (type 51600, Stoelting, Inc, IL, USA). The FLSs were isolated from the left front paw and primary cells were isolated [35]. The rat FLSs were transfected with lentiviral vectors expressing empty vector plasmid (negative control, NC), miR-146a mimic, and miR-146a inhibitor as previously described [36]. The cells in the control group were abandoned, and transfected cells were selected by growing in medium containing puromycin. After 3 days, cell suspension (20 ul) was prepared. Another 20 RA model rats were randomly classified into the control, NC, miR-146a mimic and miR-146a inhibitor groups (5 rats in each group). After successful establishment of models, the cell suspension (5 ul) was injected into the caudal vein of rats (the NC group treated with the equal amount of medium without cells). The day of injecting the FLSs was regarded as day 0. The rats were scored daily. After ten days, rats were sacrificed, and the FLSs were separated and frozen. RT-qPCR was adopted to determine the expression level of miR-146a and mRNA levels of TLR4 and NF-κB, and western blotting was performed to measure protein levels of TLR4 and NF-κB. All experiments were approved by the Ethics committee of First Teaching Hospital of Tianjin University of Traditional Chinese Medicine, and the experimental procedures were as recommended in the *Declaration of Helsinki*.

### Immunohistochemistry

Fatty tissues of each group were fixed with 40 g/L formaldehyde solution, followed by paraffin embedding, conventional dewaxing and hydration. After high-pressure antigen retrieval by citrate buffer (0.01 M), the sections were blocked with 10% goat serum at room temperature for 20 min. After the excess liquid was removed, the sections were incubated with 50 ul antibodies including PCNA (ab18197) and ICAM 1(ab171123) first at 4°C overnight and then at 37°C for 45 min. After PBS washing for 3 times (each time for 5 min), the sections were incubated with 40∼50 μl secondary antibody at 37°C for 1 h. Next, after rinsed with PBS for 3 times (each time for 5 min), the sections were stained by 3,3-diaminobenzidine (DAB) for 5∼10 min and counterstained by hematoxylin for 2 min. Then dehydration, mounting procedures and microscopic examination of the sections was performed. Cells with light yellow to brownish yellow particles in different size were determined as positive cell. Three sections were randomly selected from each specimen and 3 visual fields were selected from each section. The average absorbance of each field and the IOD of positive staining were calculated using Image-Pro Plus software (Media Cybernetics, Rockville, MD, USA).

### Statistical analysis

Data was analyzed with the SPSS18.0 statistical software (IBM Corp. Armonk, NY, USA) and presented as mean ± standard deviation. Comparisons between two groups were analyzed by *t-*test. Comparisons among multiple groups were analyzed by ANOVA, presented as percentage and rates, and analyzed by chi-square test. *P* < 0.05 indicated statistical significance.
